# Cancer history as a predictor in cardiovascular risk scores: a primary care cohort study

**DOI:** 10.3399/BJGP.2022.0088

**Published:** 2022-11-29

**Authors:** Helen Strongman, Emily Herrett, Rod Jackson, Michael Sweeting, Alexander R Lyon, Susannah Stanway, Claire Lawson, Umesh Kadam, Liam Smeeth, Krishnan Bhaskaran

**Affiliations:** Department of Non-Communicable Disease Epidemiology, London School of Hygiene and Tropical Medicine, London, UK.; Department of Non-Communicable Disease Epidemiology, London School of Hygiene and Tropical Medicine, London, UK.; Section of Epidemiology and Biostatistics, School of Population Health, University of Auckland, Auckland, New Zealand.; Department of Health Sciences, University of Leicester, Leicester, UK.; National Heart and Lung Institute, Imperial College London; Royal Brompton Hospital, London, UK.; Breast Unit, Royal Marsden Hospital, London, UK.; Department of Cardiovascular Sciences, University of Leicester, Leicester, UK.; Department of Health Sciences and Diabetes Research Centre, University of Leicester, Leicester, UK.; Department of Non-Communicable Disease Epidemiology, London School of Hygiene and Tropical Medicine, London, UK.; Department of Non-Communicable Disease Epidemiology, London School of Hygiene and Tropical Medicine, London, UK.

**Keywords:** cancer survivors, cardiovascular risk score, cohort studies, primary health care

## Abstract

**Background:**

Cardiovascular risks are raised in cancer survivors but cancer history is not included in cardiovascular risk scores that inform preventive decisions.

**Aim:**

To assess whether cancer diagnosis should be included in cardiovascular risk scores.

**Design and setting:**

Cohort study using data from English general practices linked to hospital, cancer registration, and death registration data from 1990 to 2015.

**Method:**

Adults alive 1 year after a first cancer diagnosis and age, sex, general practice, and calendar- time matched cancer-free individuals were included. Individuals with <2 years of follow-up before index, recent statin prescriptions, or pre-existing coronary heart or cerebrovascular disease were excluded. Cox proportional hazard models used to develop QRISK3 scores were replicated with added cancer history variables. Whether independent hazard ratios for these variables met thresholds for inclusion in QRISK3 (>10% relative difference with *P*<0.01) was assessed.

**Results:**

In total, 81 420 cancer survivors and 413 547 cancer-free individuals were followed for a median 5.2 years (interquartile range [IQR] 2.8– 9.1) and 6.3 years (IQR 3.5–10.2), respectively. Including a 1-year cancer survivorship variable in a QRISK3-based model met the threshold for inclusion for males (independent hazard ratio [iHR] 1.16, 95% confidence interval [CI] = 1.11 to 1.20, *P*<0.001) but not females (iHR 1.07, 95% CI = 1.01 to 1.14, *P* = 0.02). When including cancer type, the threshold was met for both sexes with history of haematological cancer (males: iHR 1.27, 95% CI = 1.16 to 1.40, *P* <0.001; females: iHR 1.59, 95% CI = 1.32 to 1.91, *P*<0.001) and for males but not females with history of solid cancers (males: iHR 1.13, 95% CI = 1.08 to 1.18, *P* <0.001; females: iHR 1.04, 95% CI = 0.98 to 1.10, *P* = 0.19).

**Conclusion:**

Developers should consider including cancer history variables in future cardiovascular risk models.

## INTRODUCTION

Improvements in the effectiveness of cancer treatment have led to a growing population of cancer survivors, with 50% of people diagnosed with cancer in high- income countries now expected to live ≥5 years.^1^ Survivors of many site-specific cancers, including haematological cancers, have elevated medium- to long-term risks of coronary heart disease and stroke compared with the general population,^2^^–^^7^ likely to be largely driven by cardiotoxicities of cancer treatments.^2^ Initiatives have been developed to prevent and mitigate cardiovascular complications of cancer therapy in cancer survivors,^8^^–^^12^ and experts have called for research investigating whether cancer should be included in cardiovascular risk scores.^13^

Widely used risk prediction equations do not take account of cancer history and may underpredict coronary heart and cerebrovascular disease in cancer survivors. In the UK, cardiovascular disease prevention measures in primary care, including statin and blood pressure-lowering prescribing, are informed by QRISK2/QRISK3 scores in accordance with the Quality and Outcomes Framework. These scores were developed using large-scale English electronic health record databases to estimate 10-year cardiovascular disease risk based on known demographic and health-related cardiovascular risk factors, but excluding cancer history.^14^^,^^15^ To the authors’ knowledge, no studies have specifically validated QRISK or other cardiovascular risk scores in cancer survivors, or formally assessed whether cancer history should be incorporated into risk prediction models.

The aim of this study was therefore to assess the predictive performance of QRISK3 scores in adult cancer survivors using large-scale electronic health records from multiple linked English databases, and ultimately evaluate whether cancer survivorship would meet criteria used to develop the risk score.^15^

## METHOD

### Design and setting

This was a retrospective cohort study using English Clinical Practice Research Datalink (CPRD) GOLD primary care data^16^ linked to Hospital Episode Statistics Admitted Patient Care (HES APC),^17^ national cancer registration,^18^ and Office for National Statistics (ONS) mortality data covering the period 1990 to 2015.

**Table:** How this fits in

Cardiovascular risk scores are used internationally by primary care physicians and GPs to inform prescribing of statins and blood pressure-lowering drugs. Although cardiovascular risks are known to be raised in cancer survivors, previous research has not formally considered whether cancer survivorship should be included in cardiovascular risk scores. The current study suggests that including 1-year cancer survivorship meets inclusion criteria for QRISK scores that are used in the UK, especially for males and haematological cancers in both sexes. Further research is needed to determine whether longer-term cancer survivorship should be included in these scores and whether novel scores should be designed specifically for cancer survivors.

CPRD collect de-identified data from participating UK general practices. Data include Read-coded clinical diagnoses, test results, and primary care prescriptions. Linked HES APC, cancer registration, and ONS mortality data include International Classification of Diseases, 10th Revision (ICD-10)-coded hospital admissions, cancer diagnoses, and causes of death.

### Participants, exposures, and outcomes

This study used data from a pre-existing cohort^2^ of 1-year survivors of the 20 most common cancers; each cancer survivor was matched to up to five cancer-free individuals on sex, age (plus or minus 3 years), and general practice.

Definitions used in the current study were Read-coded CPRD GOLD and ICD- 10-coded HES APC, and national cancer registration data. Cancers included oral cavity (C00–06), oesophageal (C15), stomach (C16), colorectal (C18–C20), liver (C22), pancreas (C25), lung (C34), malignant melanoma (C43), breast (C50), cervix (C53), uterus (C54–C55), ovarian (C56), prostate (C61), kidney (C64), bladder (C67), brain/central nervous system (C71–C72), thyroid (C73), non-Hodgkin lymphoma (C82–C85), multiple myeloma (C90), and leukaemia (C91–C95). Read and ICD-10 codes are available at https://datacompass.lshtm.ac.uk/id/eprint/1113. Incident cancer diagnoses were defined as the first code for cancer at the specified site in any of the linked databases in line with a published validation study using the same code lists.^19^ Individuals were excluded if there was <1 year of research quality follow-up in CPRD GOLD before or after cancer diagnosis.

The index date was set to 1 year after cancer diagnosis, reflecting the follow-up available in the pre-existing cohort. The cancer-free group had the same index date as the matched cancer survivor and was required to have at ≥2 years of CPRD GOLD follow-up before this date and no previous record of cancer. Individuals within a matched set who were selected as controls but were diagnosed with cancer after index could contribute as a site-specific cancer survivor (within a new set of controls) after reaching 1-year survivorship.

The outcome for the current study was coronary heart and cerebrovascular disease recorded up to 10 years after the index date identified using CPRD GOLD and HES data using Read and ICD-10 codes provided by the QRISK3 authors.^15^ As QRISK3 was developed for risk prediction among people aged 25–84 years with no prior cardiovascular disease or current statin prescriptions, people were excluded from the cancer survivor and cancer- free groups if they were aged <25 or >84 years, were diagnosed with coronary heart or cerebrovascular disease before index, or had received at least two statin prescriptions in the year before index (that is, the year after cancer diagnosis for the cancer survivor in each matched set).

The authors of the current study developed and published code lists and algorithms that use CPRD GOLD data to identify QRISK3 predictor variables with documentation describing the process and validation steps,^20^ and implemented QRISK’s published algorithm^21^ to calculate QRISK3 scores at the index date (1 year after cancer diagnosis for the cancer survivor). Index of Multiple Deprivation twentile-based groups were used in place of Townsend twentile-based groups. All available data before index were used to calculate individual variables. Missing data for smoking, body mass index, ethnicity, systolic blood pressure, standard deviation of systolic blood pressure, and total cholesterol:high-density lipoprotein cholesterol ratio were replaced with imputed values used in the published QRISK3 algorithm.^21^

### Statistical analysis

The distribution of QRISK3 predictor variables in cancer survivors and the cancer-free group are described, and the cumulative incidence of coronary heart and cerebrovascular disease over time plotted among both groups for individuals with low (<10%) and high (≥10%) QRISK3 scores, as 10% is the threshold recommended for statin initiation.^22^ Cumulative incidences were calculated both using Kaplan–Meier methods (that is, censoring at death) to match the QRISK3 approach, and allowing for death as a competing risk to explore the potential impact of accounting for the competing risk of non-cardiovascular death in future models.

#### Predictive performance of QRISK3 scores. 

The 10-year predicted and observed risks for each decile of predicted risk were plotted to assess calibration using a naïve Kaplan–Meier approach to estimate observed risks. The Harrell’s *C* statistic was used to assess discrimination; estimates were calculated using a random 10% sample because of computational limitations.

#### Addition of cancer survivorship to QRISK3 models. 

Replicating methods used to develop QRISK3 scores, Cox proportional hazards models were used to estimate the coefficients for each risk factor in males and females separately. The multiple imputation process described in Hippisley- Cox *et al*’s QRISK3 article was replicated to create five complete case datasets,^15^ and the programming code used in the current study has been published.^23^ The imputation model included all predictor variables, age interaction terms, the Nelson–Aalen estimator for the baseline cumulative hazard, and the outcome indicator. In the imputation model, continuous variables were modelled using linear regression; fractional polynomials from the published QRISK3 equation were used to model non-linear risk relations, all variables were centred using the cohort means, and variables that were not normally distributed were log transformed. The just-another- variable approach^24^ was used to include interactions with age from the QRISK3 model. Rubin’s rules were used to combine results across imputed datasets.

A binary 1-year cancer survivorship variable with an age interaction was added to the model and the hazard ratio (HR) at the mean age assessed against QRISK3’s stated criteria of adding new binary variables to the risk score if they had an independent HR of <0.90 or >1.10 and were statistically significant at the 0.01 level. The impact of adding a three-level variable and interaction incorporating cancer type (solid cancer versus haematological cancer versus cancer free) and a site-specific breakdown (20 sites versus cancer free) were assessed separately.

Statistical analyses were carried out using Stata MP (version 16).

## RESULTS

**Figure 1.**
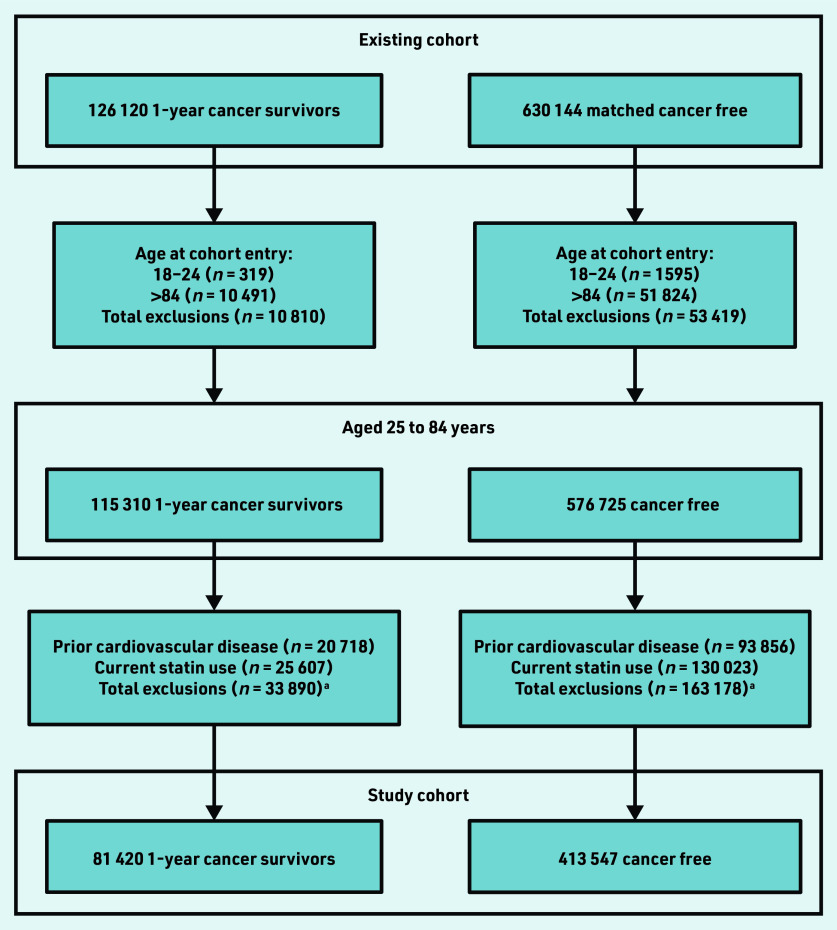
*Study cohort selection. ^a^People excluded for both prior cardiovascular disease and current statin use are included in both counts.*

In total, 81 420 1-year cancer survivors and 413 547 cancer-free individuals were identified from the current authors’ existing study cohort aged 25–84 years at cohort entry, with no prior records of coronary heart and cerebrovascular disease, and who were not current statin users (Figure 1). Cancer survivors and cancer- free individuals were followed-up for a median 5.2 years (interquartile range [IQR] 2.8–9.1) and 6.3 years (IQR 3.5–10.2), respectively. Characteristics of both groups including distributions of all QRISK3 predictors are described in Supplementary Table S1. There were 7367 (9.0%) cancer survivors who had experienced haematological cancer. Median age for both cancer survivors and cancer-free individuals at index was 62.7 (IQR 54.0–73.0) years; 47 351 (58.2%) cancer survivors and 238 697 (57.7%) cancer-free individuals were female. There were minimal differences in patient characteristics, with slightly higher proportions of missing data in the cancer- free group.

**Figure 2.**
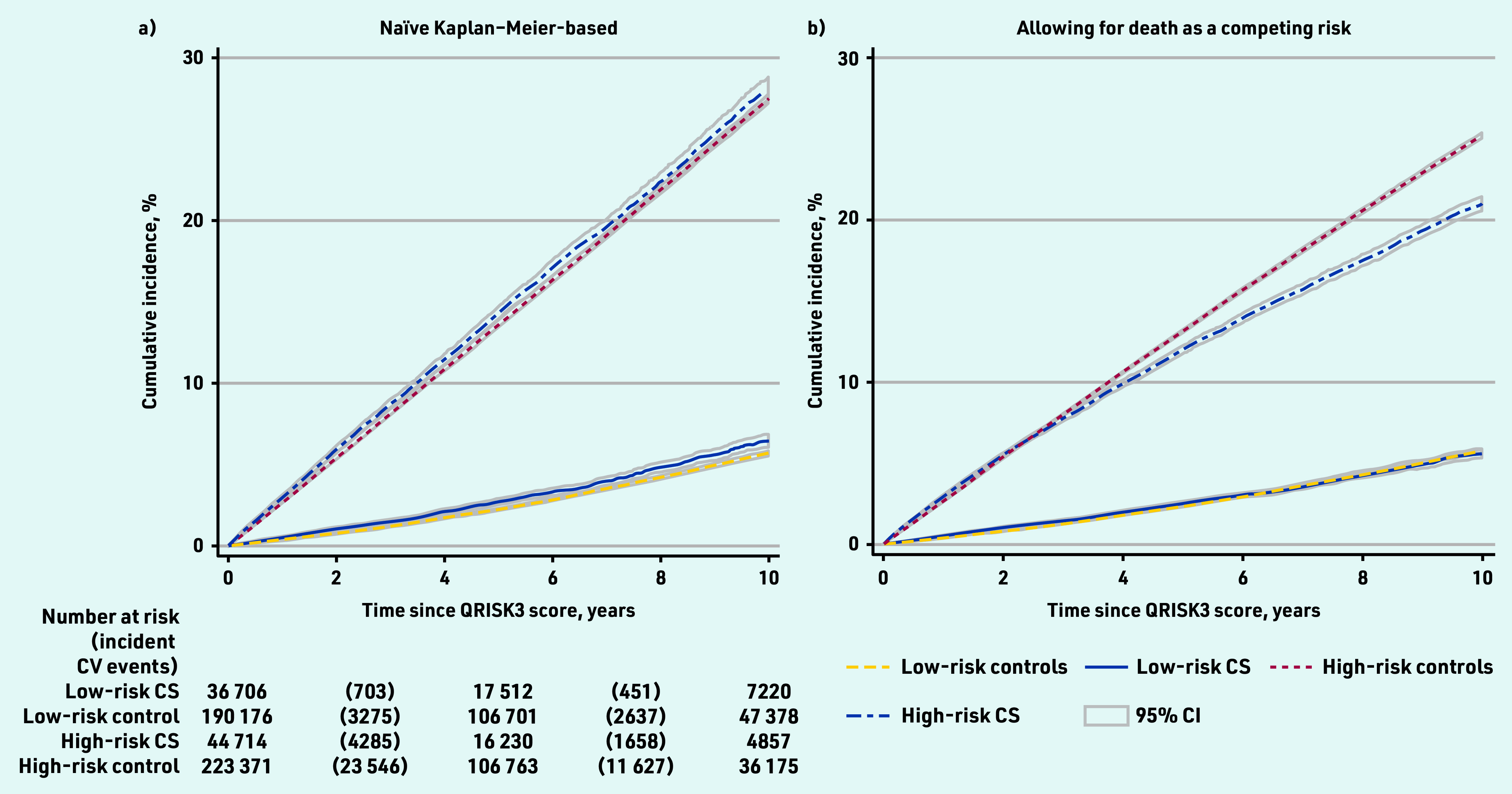
*Cumulative cardiovascular disease incidence estimated a) using Kaplan–Meier methods and b) in the presence of competing risks. Risk status determined by QRISK3 score estimated year following cancer diagnosis (≥10% = high risk). CS = cancer survivor.* *CV = cardiovascular.*

The median QRISK3 score was 11.7% (IQR 4.4–23.2) in cancer survivors and 11.3% (IQR 4.4–22.5) in the cancer-free group; 44 714 (54.9%) cancer survivors and 223 371 (54.0%) cancer-free individuals had high predicted cardiovascular risk (QRISK3 ≥10%). Over the study period, 7097 cancer survivors and 41 085 cancer-free individuals experienced a cardiovascular event. Among those with low QRISK3 scores (<10%), cumulative coronary heart and cerebrovascular disease incidence at 10 years when censoring for death was 6.4% (95% confidence interval [CI] = 6.1 to 6.9) in cancer survivors and 5.7% (95% CI = 5.5 to 5.9) in cancer-free individuals; among those with high QRISK3 scores (≥10%), 10-year cumulative incidence was 28.1% (95% CI = 27.4 to 28.8) in cancer survivors and 27.5% (95% CI = 27.2 to 27.8) in cancer- free individuals (Figure 2a).

Cumulative coronary heart and cerebrovascular disease incidence with death treated as a competing risk was similar in cancer survivors and cancer-free individuals with low coronary heart and cerebrovascular disease risk but diverged in later years in people at high cardiovascular risk because of increased mortality in cancer survivors compared with cancer- free individuals (Figure 2b).

**Figure 3.**
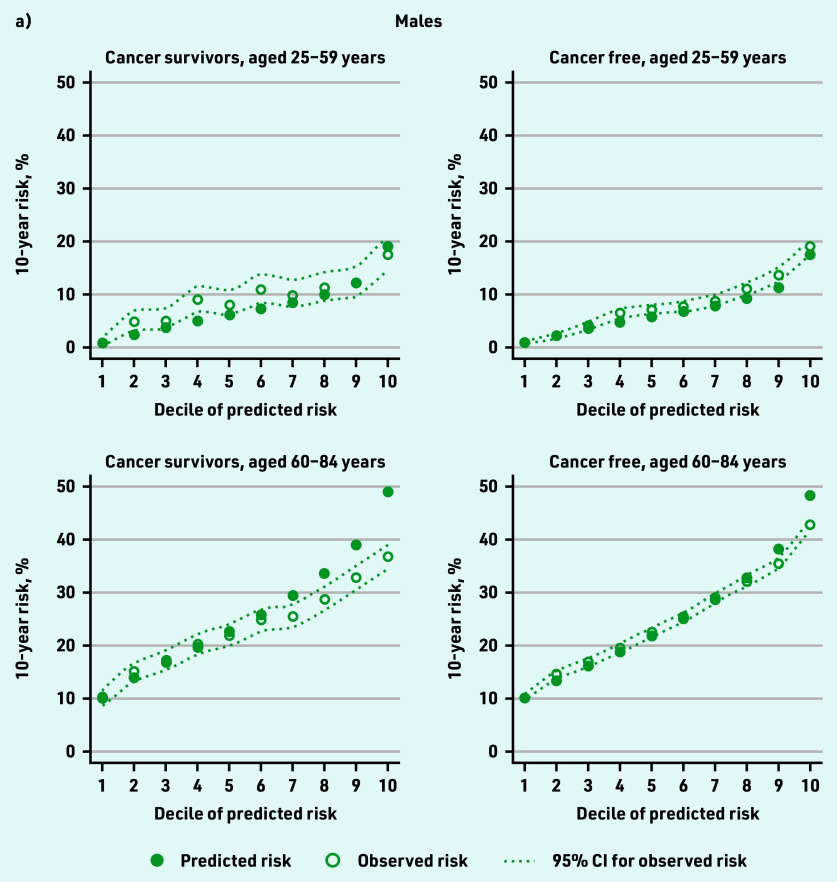

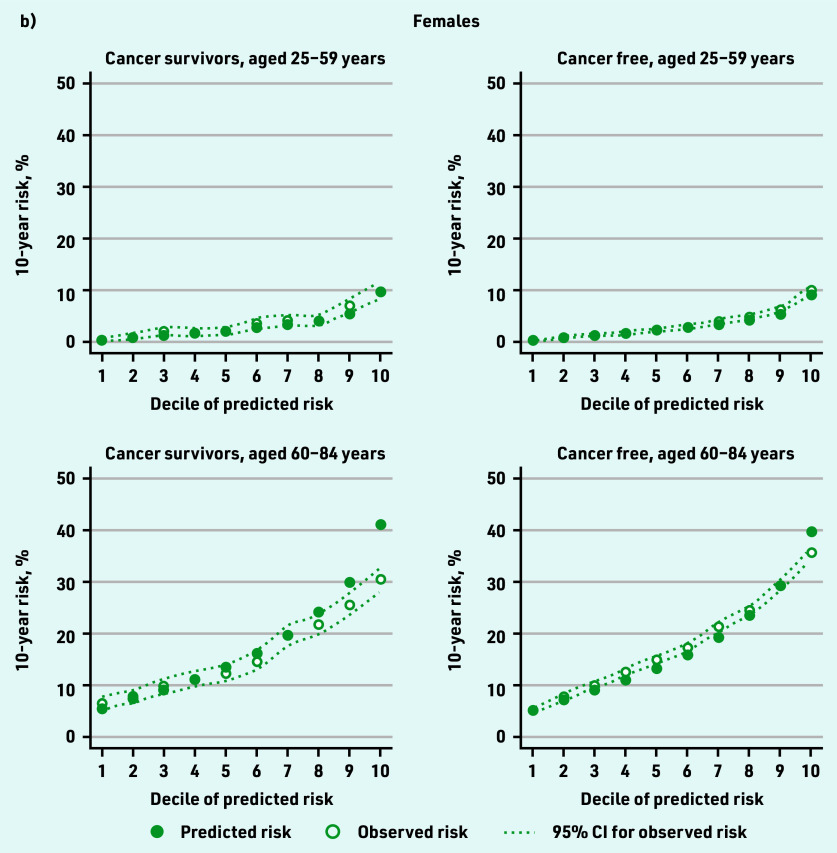
*Observed 10-year cardiovascular disease risk by decile of predicted risk for cancer survivors and cancer-free groups (calibration). a) Males and b) females.*

Figure 3 shows observed 10-year coronary heart and cerebrovascular disease risk by decile of predicted risk for cancer survivors and cancer- free individuals. One year after cancer diagnosis, there was close correspondence between predicted and observed 10-year risk in cancer survivors except for a degree of overprediction at higher deciles of risk (≤2.0% difference between predicted and observed in the lowest decile; 12.0% and 7.7% overprediction in males and females, respectively, in the highest decile). In cancer-free individuals, similar patterns were observed but with tighter calibration (<0.6% difference between predicted and observed in the lowest decile; 5.2% and 2.2% overprediction in males and females, respectively, in the highest decile). Overprediction in each group was highest in older individuals. The Harrell’s *C* statistic was 0.79 in female cancer survivors and cancer-free individuals and 0.68 in males in both groups, suggesting that the QRISK3 model has similar ability to determine which patients will have cardiovascular disease first in both groups (Table 1).

**Table 1.** Harrell’s *C* statistic comparing model discrimination in 1-year cancer survivors and the cancer-free group by age and sex

**Sex and group**	**Harrell’s *C* statistic**		

	**All ages**	**25–59 years**	**60–84 years**
**Female**			
Cancer survivor	0.79	0.74	0.66
Cancer-free group	0.79	0.70	0.68

**Male**			
Cancer survivor	0.68	0.71	0.65
Cancer-free group	0.68	0.72	0.64

**Figure 4.**
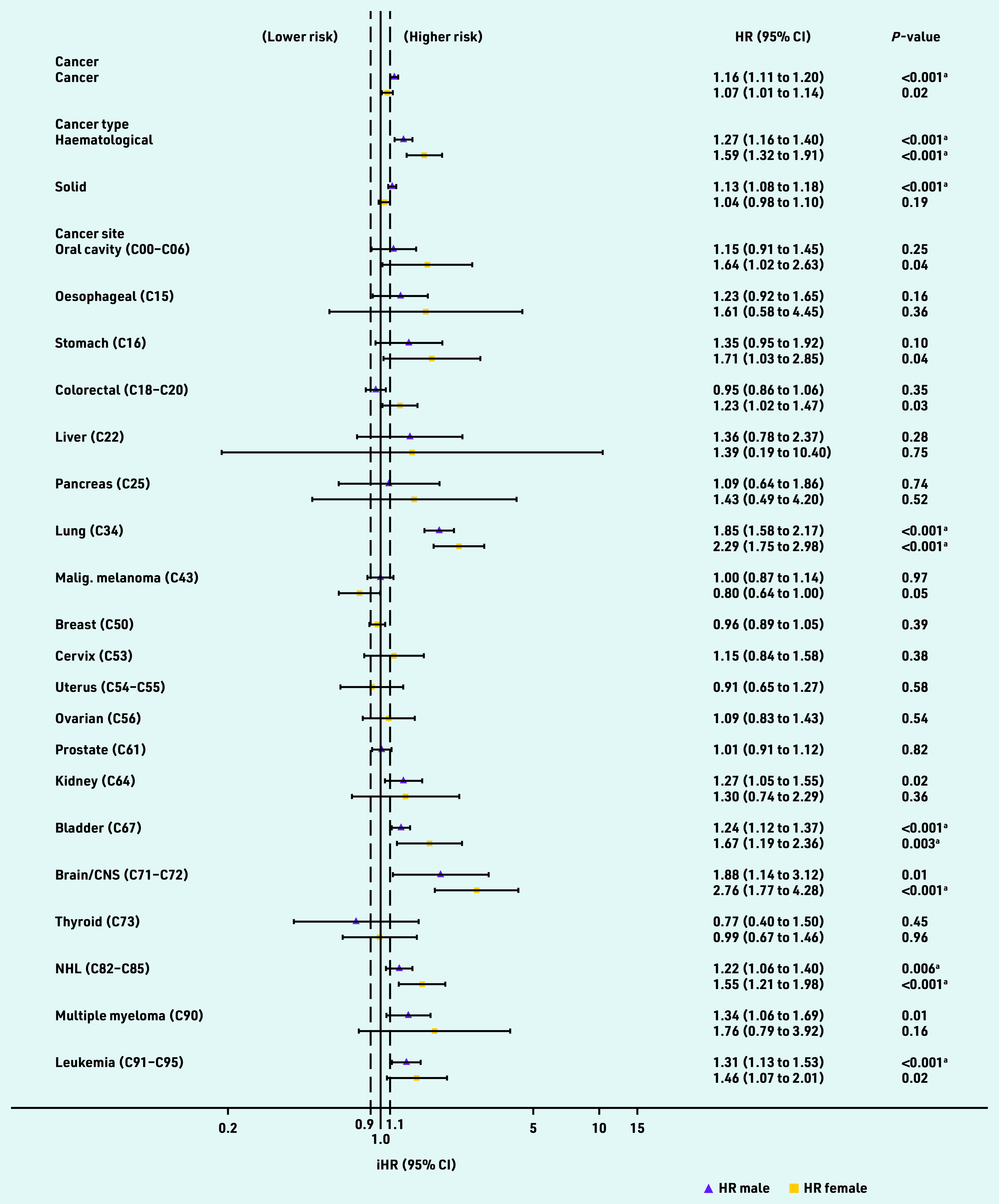
***Independent hazard ratios (HRs) for cancer variables added to QRISK3 models. HRs are evaluated at the mean age. ^a^Meets*** **P *-value threshold for inclusion in QRISK3 score. Black dashed line = HR threshold for inclusion in QRISK3 score. CNS = central nervous system. iHR = independent hazard ratio. Malig. = malignant. NHL = non-Hodgkin lymphoma.***

Figure 4 displays independent HRs for cancer survivorship variables when added to QRISK3 Cox regression models and whether they met the QRISK3 threshold for inclusion in the risk score (HR >1.1, *P*<0.01). The independent HR for 1-year cancer survivorship was 1.16 (*P*<0.001) in males and 1.07 (*P* = 0.02) in females, meeting the QRISK3 threshold in males but not females.

When solid and haematological cancers were considered separately in the model, the threshold was met for both sexes with haematological cancer (male HR 1.27, *P*<0.001; females HR 1.59, *P*<0.001) and for solid cancers in males (HR 1.13, *P*<0.001) but not females (HR 1.04, *P* = 0.19). HRs varied widely when including individual site-specific cancers in the model and confidence intervals were wide. HRs and *P*-values met the threshold for inclusion in male and female lung, bladder, and non-Hodgkin lymphoma cancer survivors, female brain cancer survivors, and male leukaemia survivors (Figure 4).

Supplementary Table S2 displays adjusted HRs for all predictor variables in the final Cox proportional hazard models. These differ from the HRs from the QRISK3 derivation models,^15^ reflecting differences in the derivation populations and the inclusion of cancer variables.

## DISCUSSION

### Summary

Including a variable for cancer history in QRISK3-based models in a population of 1-year cancer survivors and cancer-free individuals met the threshold for inclusion in the QRISK3 score for males but not females. When including a categorical variable for cancer type, the threshold was met for both males and females with haematological cancer and for males, but not females, with solid cancers.

### Strengths and limitations

A major strength of the current study was the use of similar large English primary care and linked data to those used to derive the QRISK3 score to define cancer survivorship, coronary heart and cerebrovascular disease, and predictor variables. It was therefore possible to generate comparable scores with those recorded directly in the GP data.^20^

A limitation of the study design was that, because the data source comprised a matched cohort of 1-year cancer survivors and cancer-free individuals from a previous study, follow-up was from the 1-year anniversary of cancer diagnosis for all survivors. In practice one would wish to evaluate how well models predict 10-year risk from any given time point after cancer diagnosis and estimate absolute changes in predicted risk in people who are cancer survivors and those who are cancer free in the general population; this could not be done with the data available.

The association between cancer history and cardiovascular disease risk may decrease over time since cancer diagnosis, weakening the case for inclusion in risk prediction algorithms; this will need to be evaluated by future studies. It was also not possible to include the important predictors of cardiovascular risk in cancer survivors that are not coded in general practice data, such as cancer stage and grade, chemotherapy, radiotherapy, and cardiovascular disease monitoring before and after treatment. When a categorical variable for cancer site was included in the models, CIs were wide for multiple cancer sites suggesting that the sample size was too small to meet QRISK3 criteria in some cases. Despite this, QRISK thresholds were met for male and female lung, bladder, and non-Hodgkin lymphoma cancer survivors, female brain cancer survivors, and male leukaemia survivors.

The original QRISK3 models and the current models including cancer history aim to predict coronary artery disease and stroke outcomes, but do not target prediction of cardiovascular outcomes including heart failure and venous thromboembolism, which have stronger associations with cancer survivorship.^2^ Additionally, using the QRISK’s approach of evaluating the impact at the mean age might miss predictors that have a strong impact on some age groups but not others.

### Comparison with existing literature

The descriptive findings match previous analyses reporting no differences in Framingham Risk Scores in cancer-free groups compared with survivors of breast cancer,^25^ testicular cancer,^26^ childhood cancer^27^ in the US, or ovarian cancer in Norway.^28^ However, higher 10-year Pooled Cohort Equation and Framingham Risk Scores have been observed in large cohorts of cancer survivors compared with cancer-free groups in the US^29^ and Korea,^30^ respectively. These differences may reflect variation in cancer types included in the studies, and the time since cancer diagnosis and study settings. No published studies have assessed whether cancer survivorship should be included in QRISK3 scores.

### Implications for research and practice

Ten-year risk of coronary heart and cerebrovascular disease is likely to be sufficiently elevated in 1-year cancer survivors to meet criteria for inclusion in cardiovascular risk scores, especially for haematological cancers in both sexes. This may be because the elevated risk of heart disease is the result of independent cancer- related factors, such as cardiotoxicities of chemotherapy and radiotherapy, rather than broader risk factors that are already included in QRISK3 scores.^2^

The current study supports further investigation of the benefits and limitations of including cancer survivorship variables in cardiovascular risk scores currently used in primary care settings in the UK; this could be done as part of the regular update cycles of risk score algorithms using data that are readily available in electronic health records. Future models in cohorts that are representative of the general population should explore the predictive value of cancer survivorship according to time since diagnosis, differentiating between long- and short-term cancer survivorship, and further explore the role of cancer site and cancer treatment received.

Further research is needed to determine whether new cardiovascular risk scores, specific to cancer survivors, could be developed for use in primary or secondary care to improve the health of cancer survivors and uptake of cardioprotective medications.^31^ These scores could predict a wider range of cardiovascular disease outcomes, including heart failure and venous thromboembolism, and use detailed information from specialist care about cardiovascular disease monitoring and cancer treatments that are associated with cardiovascular disease. Risk scores designed for use in specialist care before discharge should consider the importance of using competing risk models that do not censor for death to aid prioritisation between the need to cure the cancer and risk of future cardiovascular disease. Electronic transfer of clinical treatment summaries, for example, from cancer registries to general practice, may greatly improve knowledge and prediction of cardiovascular risk in this setting.^32^

## Data

Data were obtained from the Clinical Practice Research Datalink (CPRD). CPRD is a research service that provides primary care and linked data for public health research. CPRD data governance, and the authors’ own licence to use CPRD data, do not allow the authors to distribute or make available patient data directly to other parties. Researchers may apply for data access at www.cprd.com, and must have their study protocol approved via CPRD’s Research Data Governance Process (https://www.cprd.com/research-applications).

## References

[1] Cancer Research UK (2019). Cancer statistics for the UK. https://www.cancerresearchuk.org/health-professional/cancer-statistics-for-the-uk.

[2] Strongman H, Gadd S, Matthews A (2019). Medium and long-term risks of specific cardiovascular diseases in survivors of 20 adult cancers: a population-based cohort study using multiple linked UK electronic health records databases. Lancet.

[3] Armenian SH, Xu L, Ky B (2016). Cardiovascular disease among survivors of adult-onset cancer: a community-based retrospective cohort study. J Clin Oncol.

[4] Zhang F, Wang K, Du P (2021). Risk of stroke in cancer survivors: a meta-analysis of population-based cohort studies. Neurology.

[5] Kero AE, Järvelä LS, Arola M (2014). Cardiovascular morbidity in long-term survivors of early-onset cancer: a population-based study. Int J Cancer.

[6] Rugbjerg K, Mellemkjaer L, Boice JD (2014). Cardiovascular disease in survivors of adolescent and young adult cancer: a Danish cohort study, 1943–2009. J Natl Cancer Inst.

[7] Henson KE, Reulen RC, Winter DL (2016). Cardiac mortality among 200 000 five-year survivors of cancer diagnosed at 15 to 39 years of age: the teenage and young adult cancer survivor study. Circulation.

[8] Zamorano JL, Lancellotti P, Rodriguez Munoz D (2016). 2016 ESC Position Paper on cancer treatments and cardiovascular toxicity developed under the auspices of the ESC Committee for Practice Guidelines: the task force for cancer treatments and cardiovascular toxicity of the European Society of Cardiology (ESC). Eur Heart J.

[9] Chang H-M, Okwuosa TM, Scarabelli T (2017). Cardiovascular complications of cancer therapy: best practices in diagnosis, prevention, and management: part 2. J Am Coll Cardiol.

[10] Chang H-M, Moudgil R, Scarabelli T (2017). Cardiovascular complications of cancer therapy: best practices in diagnosis, prevention, and management: part 1. J Am Coll Cardiol.

[11] Virani SA, Dent S, Brezden-Masley C (2016). Canadian Cardiovascular Society guidelines for evaluation and management of cardiovascular complications of cancer therapy. Can J Cardiol.

[12] Mehta LS, Watson KE, Barac A (2018). Cardiovascular disease and breast cancer: where these entities intersect: a scientific statement from the American Heart Association. Circulation.

[13] Blaes AH, Shenoy C (2019). Is it time to include cancer in cardiovascular risk prediction tools?. Lancet.

[14] Hippisley-Cox J, Coupland C, Vinogradova Y (2008). Predicting cardiovascular risk in England and Wales: prospective derivation and validation of QRISK2. BMJ.

[15] Hippisley-Cox J, Coupland C, Brindle P (2017). Development and validation of QRISK3 risk prediction algorithms to estimate future risk of cardiovascular disease: prospective cohort study. BMJ.

[16] Herrett E, Gallagher AM, Bhaskaran K (2015). Data resource profile: Clinical Practice Research Datalink (CPRD). Int J Epidemiol.

[17] Herbert A, Wijlaars L, Zylbersztejn A (2017). Data resource profile: Hospital Episode Statistics Admitted Patient Care (HES APC). Int J Epidemiol.

[18] Henson KE, Elliss-Brookes L, Coupland VH (2019). Data resource profile: National Cancer Registration Dataset in England. Int J Epidemiol.

[19] Strongman H, Williams R, Bhaskaran K (2020). What are the implications of using individual and combined sources of routinely collected data to identify and characterise incident site-specific cancers? a concordance and validation study using linked English electronic health records data. BMJ Open.

[20] Gadd S, Herrett E, Strongman H (2022). emilyherrett/qrisk_cprd_gold: QRISK bundle version 2.0, code lists added in version 5.0. https://zenodo.org/record/3981238.

[21] ClinRisk Ltd (2017). QRISK3-2017. https://qrisk.org/three/src.php.

[22] National Clinical Guidance Centre (2016). Cardiovascular disease: risk assessment and reduction, including lipid modification CG181.

[23] Strongman H (2022). emilyherrett/qrisk_cprd_gold: multiple imputation do file added.

[24] White IR, Royston P, Wood AM (2011). Multiple imputation using chained equations: issues and guidance for practice. Stat Med.

[25] Anderson C, Nichols HB, Deal AM (2018). Changes in cardiovascular disease risk and risk factors among women with and without breast cancer. Cancer.

[26] Feldman DR, Ardeshir-Rouhani-Fard S, Monahan P (2018). Predicting cardiovascular disease among testicular cancer survivors after modern cisplatin-based chemotherapy: application of the Framingham Risk Score. Clin Genitourin Cancer.

[27] Landy DC, Miller TL, Lopez-Mitnik G (2012). Aggregating traditional cardiovascular disease risk factors to assess the cardiometabolic health of childhood cancer survivors: an analysis from the Cardiac Risk Factors in Childhood Cancer Survivors Study. Am Heart J.

[28] Liavaag AH, Tonstad S, Pripp AH (2009). Prevalence and determinants of metabolic syndrome and elevated Framingham risk score in epithelial ovarian cancer survivors: a controlled observational study. Int J Gynecol Cancer.

[29] Zhang X, Pawlikowski M, Olivo-Marston S (2021). Ten-year cardiovascular risk among cancer survivors: the National Health and Nutrition Examination Survey. PLoS One.

[30] So JH, Lee JK, Shin JY, Park W (2016). Risk of cardiovascular disease using Framingham Risk Score in Korean cancer survivors. Korean J Fam Med.

[31] Chidwick K, Strongman H, Matthews A (2018). Statin use in cancer survivors versus the general population: cohort study using primary care data from the UK clinical practice research datalink. BMC Cancer.

[32] Walter FM, Usher-Smith JA, Yadlapalli S, Watson E (2015). Caring for people living with, and beyond, cancer: an online survey of GPs in England. Br J Gen Pract.

